# Macrophages and microglia in glioblastoma: heterogeneity, plasticity, and therapy

**DOI:** 10.1172/JCI163446

**Published:** 2023-01-03

**Authors:** Fatima Khan, Lizhi Pang, Madeline Dunterman, Maciej S. Lesniak, Amy B. Heimberger, Peiwen Chen

**Affiliations:** Department of Neurological Surgery, Lou and Jean Malnati Brain Tumor Institute, Robert H. Lurie Comprehensive Cancer Center, Feinberg School of Medicine, Northwestern University, Chicago, Illinois, USA.

## Abstract

Glioblastoma (GBM) is the most aggressive tumor in the central nervous system and contains a highly immunosuppressive tumor microenvironment (TME). Tumor-associated macrophages and microglia (TAMs) are a dominant population of immune cells in the GBM TME that contribute to most GBM hallmarks, including immunosuppression. The understanding of TAMs in GBM has been limited by the lack of powerful tools to characterize them. However, recent progress on single-cell technologies offers an opportunity to precisely characterize TAMs at the single-cell level and identify new TAM subpopulations with specific tumor-modulatory functions in GBM. In this Review, we discuss TAM heterogeneity and plasticity in the TME and summarize current TAM-targeted therapeutic potential in GBM. We anticipate that the use of single-cell technologies followed by functional studies will accelerate the development of novel and effective TAM-targeted therapeutics for GBM patients.

## Introduction

Glioblastoma (GBM) is the most common and aggressive type of primary adult malignant tumor in the central nervous system (CNS) and accounts for about 33% of all CNS tumors (1). Owing to marked tumor heterogeneity, GBM is a challenging cancer to treat (2). Genomic profiling has identified several key signaling pathways in GBM, which motivated clinical trials to test targeted therapies. Unfortunately, these efforts were unsuccessful because of glioma cell heterogeneity, which ensures the survival of cell subpopulations irrespective of treatments (3–5). Based on tumor-intrinsic gene expression profiles, GBM tumors are classified into three transcriptional subtypes (proneural, mesenchymal, and classical), with each subtype harboring different levels of tumor microenvironment (TME) heterogeneity (6, 7). In addition to cell-autonomous mechanisms, the signaling of cancer cells extends to the TME (8–10). Reciprocally, the TME can promote GBM progression and induce resistance to chemotherapy (11) and immunotherapy (12–14). Despite potent antitumor effects that have been observed in multiple cancer types (15, 16), immunotherapies such as immune checkpoint inhibitors only produce minor clinical benefits in GBM, partially because of the immunosuppressive TME (12, 17). Increasing evidence shows that immunosuppression in GBM is triggered by a symbiotic interaction between glioma cells and the TME (12, 14, 18). Therefore, targeting this symbiosis is a promising strategy to improve the antitumor efficiency of immunotherapies in GBM (12). Together, these findings highlight the role of tumor heterogeneity (including inter- and intratumor heterogeneity) in GBM progression and therapy resistance.

Among the TME components, tumor-associated macrophages and microglia (TAMs) are the most abundant population of immune cells, accounting for up to 50% of total live cells in the whole GBM tumor mass (19). Emerging evidence demonstrates that TAMs are critical for promoting tumor progression and inducing immunosuppression in GBM (20). However, therapeutic strategies for depleting TAMs have not been well translated into the clinic (21), suggesting that our understanding of this cell population is still limited. The recent development of single-cell technologies such as single-cell RNA sequencing (scRNA-Seq) and cytometry by time of flight (CyTOF) has facilitated the understanding of TAM heterogeneity in GBM (22, 23). These developments have revealed context-dependent therapeutic potential for targeting specific TAM subpopulations and/or functional states. In this Review, we discuss the origin, heterogeneity, phenotypes, and functional plasticity of TAMs in GBM. Moreover, we pinpoint the aspects of emerging single-cell technologies to identify new TAM subpopulations, which might play a critical role in GBM progression and immunosuppression. Finally, we discuss the current TAM-targeted therapeutic potential in GBM.

## TAM origin, identity, and heterogeneity

**Figure 1:**
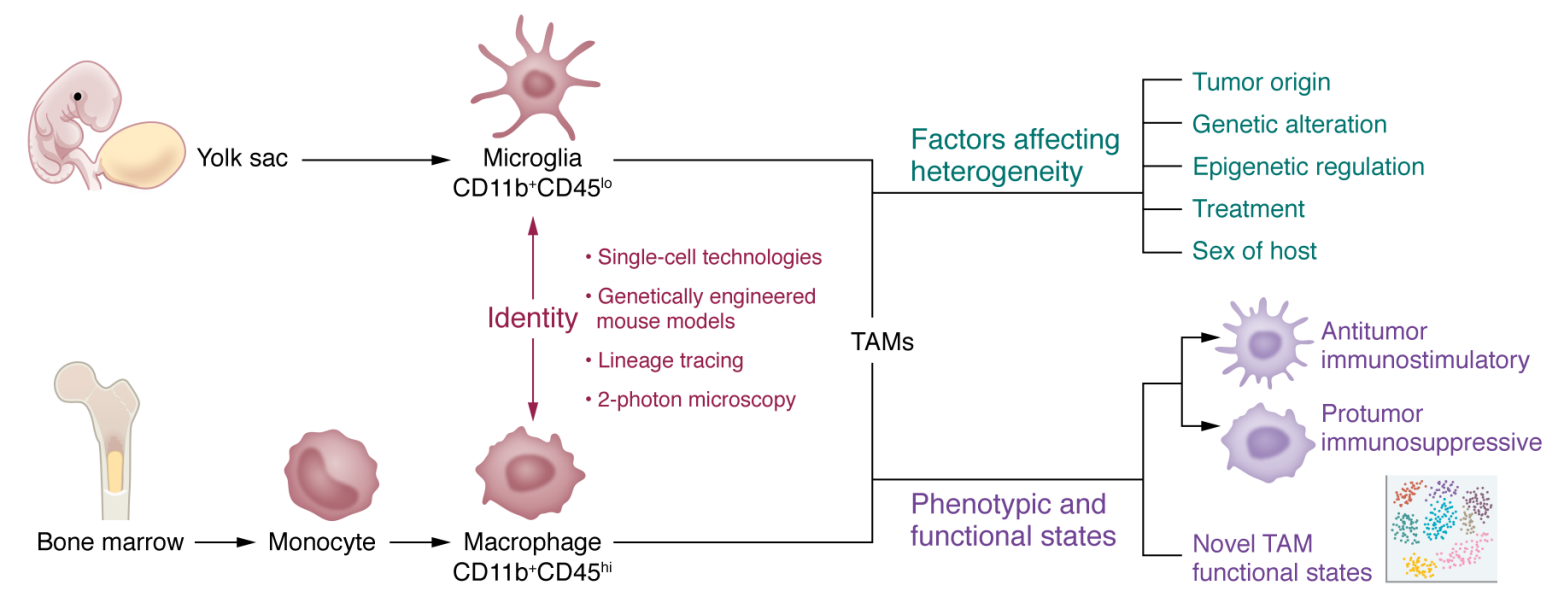
TAM origin, identity, and heterogeneity in GBM. TAMs in GBM include brain-resident microglia and macrophages that arise from the yolk sac and bone marrow and can be characterized as CD11b^+^CD45^lo^ and CD11b^+^CD45^hi^ cells, respectively. In addition to specific markers, microglia can be distinguished from macrophages using advanced approaches (e.g., single-cell technologies, genetically engineered mouse models, lineage tracing, and intravital two-photon microscopy). TAM heterogeneity is regulated in a context-dependent manner (e.g., distinct tumor origins, genetic and epigenetic alterations, treatments, and sex of the host). TAMs are typically characterized as immunostimulatory (antitumor) and immunosuppressive (protumor) phenotypes. However, single-cell technology development expands our understanding of this plasticity in GBM.

TAMs in GBM are composed of bone marrow–derived macrophages (BMDMs; hereafter referred to as macrophages) and brain-resident microglia (hereafter referred to as microglia) that originate from progenitor cells in the bone marrow and embryonic yolk sac, respectively (9, 19). In general, macrophages can be distinguished from microglia using specific cell surface markers and advanced tools (Figure 1). Subtle differences in CD45 protein expression have previously been used to distinguish between CD11b^+^CD45^hi^ macrophages and CD11b^+^CD45^lo^ microglia in GBM tumors from mouse models (24, 25). However, this classification has limitations, because the expression of CD45 in microglia can be upregulated under certain pathological conditions, including in the GBM TME (26–28). With the development of single-cell technologies, more markers have been identified to distinguish these two populations. For example, CyTOF, scRNA-Seq, and cellular indexing of transcriptomes and epitopes by sequencing (CITE-seq) analyses have demonstrated that *CCR2*, *CD45RA*, *CD141*, *ICAM*, *CD1C*, *CD1B*, *TGFBI*, *FXYD5*, *FCGR2B*, *CLEC12A*, *CLEC10A*, *CD207*, *CD49D*, and *CD209* are more likely enriched in macrophages, whereas *CX3CR1*, *SALL1*, *HEXB*, *P2RY12*, and *TMEM119* are highly expressed in microglia (23, 24, 29–34). Since TAMs are highly plastic, integration of multiple markers is required to distinguish macrophages from microglia in the GBM TME. Moreover, recent studies using advanced approaches (e.g., genetically engineered mouse models, genetic lineage tracing, and intravital two-photon microscopy) have made further progress in determining macrophage and microglia identity. For example, the ontogeny of these two populations has been suggested in GBM tumors established in *Cx3cr1^CreER^ R26^YFP^* mice (24) and *Cx3cr1^GFP/WT^ Ccr2^RFP/WT^* knockin mice (35). Genetic lineage tracing studies have nominated CD49D as a macrophage marker in GBM tumors (36). Moreover, studies using intravital two-photon microscopy revealed that macrophages and microglia in GBM tumors have morphological and behavioral differences (37). Microglia are highly branched stationary cells with larger cell sizes, whereas macrophages have a better migratory ability with fewer branches and smaller sizes (37). Given these differences, we hypothesize that monocytes may take advantage of their morphological features to cross the blood-brain barrier and then differentiate into macrophages in the TME, whereas infiltrating microglia can quickly change their states (e.g., exhibiting downregulation of homeostatic genes and upregulation of IFN and phagocytic/lipid signatures) during tumor development (24). However, further studies are needed to decipher how these morphological differences between macrophages and microglia affect their functions and dynamics in the GBM TME.

TAMs in brain cancers are a population of heterogeneous immune cells. Single-cell analyses of brain tumors demonstrate that TAM compositions in primary brain tumors differ from those in metastatic brain tumors (tumors originate from other locations in the body, such as breast and lung) (18, 23). Specifically, primary brain tumor (e.g., GBM) is more likely to be infiltrated with reactive microglia (CD49d^–^Mertk^+^CX3CR1^+^CD11c^+^CD64^+^ cells). These microglia are diffusely scattered throughout GBM tumor regions but are absent from the core of metastatic brain tumors (23). In contrast, macrophages localize near CD31^+^ vascular structures in GBM and brain metastatic tumors (19, 23). In addition, single-cell analyses have provided further evidence supporting TAM heterogeneity in GBM patient tumors (24, 38). For example, multiple TAM subsets with distinct gene signatures, such as macrophage in the transitory state (with high *LYZ*, *EREG*, and *S100A6* expression and low *C1Q* expression), microglia-like macrophage (with high *BIN1*, *CX3CR1*, *TMEM119*, and *OLFML3* expression), hypoxic macrophage (with high *BNIP3*, *ADAM8*, *FAM162A*, and *MIF* expression), and phagocytic/lipid macrophage (with high *FABP5*, *GPNMB*, *LGALS3*, and *CD63* expression), have been identified in GBM patient and mouse tumors (24). Growing evidence further supports that the heterogeneity of TAMs in GBM is context dependent (19). First, tumor origin (e.g., newly diagnosed tumor versus recurrent tumor) is a prominent factor contributing to this heterogeneity (Figure 1). It has been shown that microglia are the predominant cell population in newly diagnosed GBM tumors, whereas macrophages outnumber microglia in recurrent GBM tumors (24, 38).

The genetic alterations in GBM also affect TAM heterogeneity (Figure 1). Genetic profiling of GBM tumors has identified key mutational genes (e.g., *TP53*, *EGFR*, *NF1*, and *PTEN*) (39). Since distinct immune cell compositions are observed in different GBM subtypes, one hypothesis is that these genetic alterations contribute to immunological changes in the TME. Indeed, TAM infiltration is significantly triggered by the mutation/deletion of *NF1* and *PTEN* in mesenchymal GBM (7, 40). Another frequently mutated gene in gliomas (generally low grade) is isocitrate dehydrogenase 1 (*IDH1*), a key enzyme that regulates tryptophan metabolism (30). Gliomas mutant for *IDH* have a better prognosis than IDH–wild type (WT) tumors (typically grade IV GBM) (41). Compared with *IDH*-mutant tumors, *IDH*-WT GBM tumors harbor microglia with increased expression of reactive phenotype genes (e.g., CD14 and CD64) and more macrophages with increased expression of HLA-DR and MHC I/II genes (18). Mechanistically, *IDH* mutation reduces the differentiation of monocytes toward macrophages, which is supported by the CyTOF data showing that *IDH*-WT and *IDH*-mutant tumors are enriched with CD163^+^CX3CR1^+^CADM1^+^ macrophages and CD33^+^CCR2^+^CD14^+^ undifferentiated monocytes, respectively (23). Additionally, the heterogeneity of TAMs relates to the tumor stage in gliomas under specific genetic backgrounds. A recent study using scRNA-Seq and CyTOF technologies in a mouse model demonstrated that *IDH*-mutant tumors harbor more microglia but fewer macrophages than *IDH*-WT tumors at the early stage (30). However, macrophage infiltration was increased in the *IDH*-mutant mouse model rather than in the *IDH*-WT counterpart during the tumor progression (30). Despite distinct transcriptional profiles and dynamics, macrophages and microglia in *IDH*-WT GBM display a similar expression pattern of genes (e.g., *THBS1*, *TGFBI*, *FN1*, and *VCAN*) that regulate extracellular matrix proteins (18), suggesting that different TAM subpopulations may cooperatively shape the TME in response to a certain genetic mutation. As a result of their infiltration, macrophages maintain GBM cells and/or glioma stem cells (GSCs) in a mesenchymal subtype by secreting innate immunity–associated cytokines such as TNF-α (42), whereas microglia display such an effect via remodeling of metabolic transcriptomes (e.g., SREBP1/2) and the nitric oxide synthesis pathway (42). From these findings, we conclude that the heterogeneity of TAMs relates to genetic alterations (e.g., *NF1*, *PTEN*, and *IDH1* deletion and/or mutation) of glioma cells.

Furthermore, epigenetic regulation of glioma cells contributes to TAM heterogeneity (Figure 1). A recent study demonstrates that the infiltration of PD-L1^+^ macrophages in GBM tumors is triggered by epigenetic changes of GSCs following an immune attack but is independent of genetic selection (43). Moreover, recent studies focusing on epigenetic regulator screen have identified circadian locomotor output cycles protein kaput (CLOCK) as a top hit in GSCs that promotes the infiltration of microglia, but not macrophages, in GBM (25, 44). Finally, the TAM heterogeneity is also manifested in GBM upon treatments (e.g., the standard of care) and regulated in a sex-specific manner. For example, ionizing radiation shifts the TAM composition toward macrophages rather than microglia in recurrent GBM, suggesting macrophages as a key target for GBM recurrence after radiation therapy (29). Microglia from male GBM mouse models and patients express more MHC II components (e.g., *H2-Aa*, *H2-Ab1*, *H2-Eb*) and *Cd74* than microglia from female GBM (33). Taken together, these findings indicate that TAMs are a highly heterogeneous population of cells, and that this heterogeneity is context dependent (e.g., tumor under specific origin, genetic and epigenetic backgrounds, treatment, and sex of the host) in GBM.

## TAM phenotype and function

Macrophage phenotypic and functional plasticity has been a robust topic of debate in the neuroimmunology field (45). The M1/M2 dichotomy was proposed to classify “classically activated” and “alternatively activated” macrophages, respectively (46). M1 macrophages are induced by exposure to proinflammatory cytokines (e.g., IFN-γ and TNF-α), which, in turn, eliminate tumor cells by producing inflammatory factors, such as reactive oxygen species (ROS), TNF-α, IL-1β, IL-6, IL-12, and IL-23 (19, 47). On the other hand, M2 polarization is triggered upon stimulation with antiinflammatory cytokines (e.g., IL-4, IL-10, or IL-13), and such polarized macrophages show less cytotoxicity on tumor cells via production of antiinflammatory cytokines, e.g., IL-10, TGF-β, CCL22, and CCL17 (48–50). While it has been initially useful for macrophage categorization, increasing evidence demonstrates that the M1/M2 dichotomy faces several challenges. First, the dichotomy is proposed primarily based on the results from in vitro studies (51), and this phenomenon is not well manifested in vivo as demonstrated by either extensive immune phenotyping or scRNA-Seq studies in GBM patient tumors (38, 52). As such, there is no identifiable correlation between in vitro–defined macrophage phenotype markers and macrophage cluster signature genes identified in GBM patient tumors (38, 52). However, it should be noted that a specific subpopulation of TAMs in GBM patient tumors harbor a glucocorticoid-induced signature (24), consistent with the in vitro studies in macrophages (53). Second, it is overly simplistic to define TAM phenotypes solely based on a selection of markers (54). Although some markers (e.g., CD163 and CD206) have been identified in an attempt to distinguish the M1/M2 phenotype in TAMs (54, 55), specific markers for M1 or M2 macrophages are still missing (46, 56). Third, TAMs do not display bona fide M1 or M2 phenotype, but, rather, likely exist in a continuum or a less differentiated state. For instance, single-cell profiling on human glioma tumors has demonstrated that TAMs coexpress M1 and M2 genes in individual cells (52). Finally, TAM phenotypes are dynamic and fluid, and respond to distinct TME and/or therapeutic intervention (19). Together, these findings suggest a complexity of TAMs in GBM that cannot be fully understood based solely on the M1/M2 dichotomy.

Single-cell technologies are increasingly used to characterize the phenotypic and functional plasticity of TAMs in GBM (Figure 1). The results of these studies not only support that TAM activation within the GBM TME may not follow the M1/M2 dichotomy (38, 52), but also illustrate novel TAM phenotypic and functional states. For example, scRNA-Seq analysis of normal mouse brains and tumors from the GL261 mouse model demonstrates that the macrophage subpopulation expressing *Ccl22*, *Cd274* (encoding PD-L1), and *Ccl5* supports an immunosuppressive functional state (33). Similarly, single-cell profiling of human GBM tumors has identified a novel macrophage versus microglia functional state, where macrophages show upregulated immunosuppressive cytokines and activated tricarboxylic acid cycle (52). In an additional scRNA-Seq study, lineage markers of individual cells in each cluster were used to classify the molecular subtypes of myeloid cells in GBM (38). Among the nine identified molecular subtypes, two clusters are macrophages with distinct functional states displayed. One cluster of macrophages are immunosuppressive cells, and the other cluster of cells are proliferating macrophages enriched with classical inflammatory hallmarks (38). To conclude, single-cell analysis in GBM tumors is increasingly changing our understanding of TAM phenotypes and functional plasticity, which represents an exciting opportunity to develop personalized therapeutic strategies by targeting specific TAM states in GBM patients.

## Newly identified TAM subpopulations

**Figure 2:**
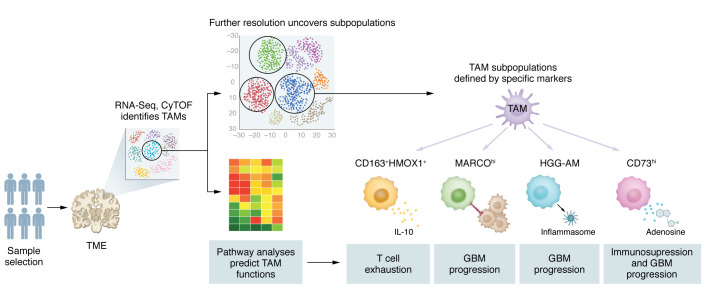
Identification of new TAM subpopulations in GBM. The understanding of TAM heterogeneity and functional plasticity in the GBM TME is expanding with the development of single-cell technologies (e.g., scRNA-Seq and CyTOF). Unbiased pathway analyses followed by functional validations would help characterize context-dependent TAM functions. Notably, quite a few TAM subpopulations and their potential biological functions have been identified and deciphered (as indicated). CD73, cluster of differentiation 73; CyTOF, cytometry by time of flight; GBM, glioblastoma; HGG-AM, high-grade glioma–associated microglia; HMOX1, heme oxygenase 1; MARCO, macrophage receptor with collagenous structure; scRNA-Seq, single-cell RNA sequencing; TAM, tumor-associated macrophage; TME, tumor microenvironment.

Compared with other methods, single-cell technologies have a unique advantage in identifying rare or previously unknown TAM subpopulations, which might be critical for GBM progression and immunosuppression (Figure 2). For instance, a recent scRNA-Seq study of a de novo GBM mouse model with human *EGFR* overexpression and loss of *Cdkn2a* and *Pten* identified four clusters of macrophages, including one cluster of perivascular immunosuppressive macrophages with high expression of *Cd163* and *Mrc1* and three clusters of microglia (57). Among the three microglial clusters, one new population of Ki67^+^ proliferative microglia expressed high levels of genes related to the G_2_/M and S phases of the cell cycle. Bulk RNA-Seq analysis on proliferating microglia demonstrates that the population of proliferative microglia commit less to the polarization program (57). An additional scRNA-Seq analysis on GL261 tumors showed that GBM tumors harbor two other microglia populations compared with the naive brains of mice (33). One such population of microglia expressed genes encoding MHC I (e.g., *H2-D1*, *H2-K1*, and *B2m*) and MHC II (e.g., *H2-Oa* and *H2-DMa*), and the other microglial population expressed genes related to cell proliferation (e.g., *Cdk1*, *Stmn1*, *Tuba1b*, *Tubb5*, and *Top2a*) (33). Transcriptional network analysis further demonstrated that MHC II–high active microglia express more chemokine-encoding genes (e.g., *Ccl3*, *Ccl4*, and *Ccl12*) (33), suggesting that this subset of microglia may help to recruit other immune cells. Conversely, macrophages have higher *Cd274* expression than microglia, thus displaying a more robust immunosuppressive function (33). Similarly, scRNA-Seq analysis in GBM patient tumors resulted in identification of a new population of proinflammatory and proliferative microglia (58) and a new population of immunosuppressive CD163^+^HMOX1^+^ microglia, which induce T cell exhaustion via release of IL-10 (59). Further scRNA-Seq analyses in mouse and human GBM tumors demonstrate that certain mouse models might not be able to fully recapitulate the functional heterogeneity of TAMs observed in GBM patients (24, 60). For example, *Cst*, *Hexb*, and *Sparc* are highly differentially expressed between microglia and macrophages in mouse tumors but not in human tumors. In contrast, *APOC2*, *TMIGD3*, and *SCIN* are microglia-specific markers restricted to human tumors (24).

Emerging evidence demonstrates that various TAM subpopulations may infiltrate into specific subtypes of GBM tumors, which result in a context-dependent symbiotic interaction between distinct TAM subpopulations and glioma cells in the TME. Here, we summarize three strategies to identify new and context-dependent TAM subpopulations in GBM. The first strategy is to perform single-cell analyses in GBM tumors comparing different molecular subtypes. For example, in human GBM ex vivo organotypic tissue culture model and primary GBM specimens, MARCO^hi^ macrophages and CD163^+^HMOX1^+^ microglia have been identified solely in mesenchymal GBM tumors (59, 61). MARCO^hi^ macrophages have been shown to promote mesenchymal transition in vitro and in vivo. Coimplantation of GSCs and MARCO^hi^ macrophages significantly decreases the survival of tumor-bearing mice (61). Functionally, HMOX1^+^ microglia distribute in the interface between GBM cells and T cells to drive T cell exhaustion (59). Although scRNA-Seq data unmask the transcriptional and spatial correlations between HMOX1^+^ microglia and mesenchymal-like GBM (59), further functional validation is needed to validate whether HMOX1^+^ microglia can shift GBM cells toward a mesenchymal-like state. The second strategy is to identify novel TAM subpopulations under specific GBM genetic backgrounds. For example, scRNA-Seq analyses on GBM patient tumors resulted in identification of a subset of high-grade glioma–associated microglia (HGG-AM) in *IDH1*-WT/SETD2-mutant GBM (58). HGG-AM are proinflammatory and proliferative cells that can promote GBM progression by inducing apolipoprotein E–mediated NLRP1 inflammasome formation (58). The third strategy is to compare the immune profile of GBM tumors that have differentially responded to treatment. This strategy can help to identify TAM subpopulations responsible for resistance development following therapies such as immune checkpoint inhibitors (ICIs). Deep immune profiling of ICI-responsive and ICI-refractory mouse models using CyTOF demonstrated that ICI-refractory GBM is associated with the accumulation of PD-L1^+^ TAMs and lack of MHC II^+^ antigen-presenting cells (20). It is worth noting that multiple TAM subpopulations likely drive the immune evasion of GBM. In addition to PD-L1^+^ TAMs, scRNA-Seq and CyTOF analyses reveal that CD73^hi^ macrophages are immunosuppressive cells and have a signature distinct from microglia that persist after anti–PD-1 treatment (62). Mechanistically, CD73^hi^ macrophages do not directly impact T cell effector responses. Rather, knocking out CD73 decreases immunosuppressive CD206^+^Arg1^+^VISTA^+^PD-1^+^CD115^+^ myeloid cells and increases iNOS^+^ myeloid cells, which, in turn, enhances the antitumor efficiency of ICI (e.g., anti–PD-1 and anti-CTLA4) therapies in murine glioma (62). Together, these findings suggest that single-cell technologies are decisive for identifying novel TAM subpopulations in GBM under specific contexts, which may pave the way for the development of context-dependent therapeutic strategies via targeting of distinct TAM subpopulations alone or in combination with immunotherapies.

## Therapeutic potential to target TAMs

**Figure 3:**
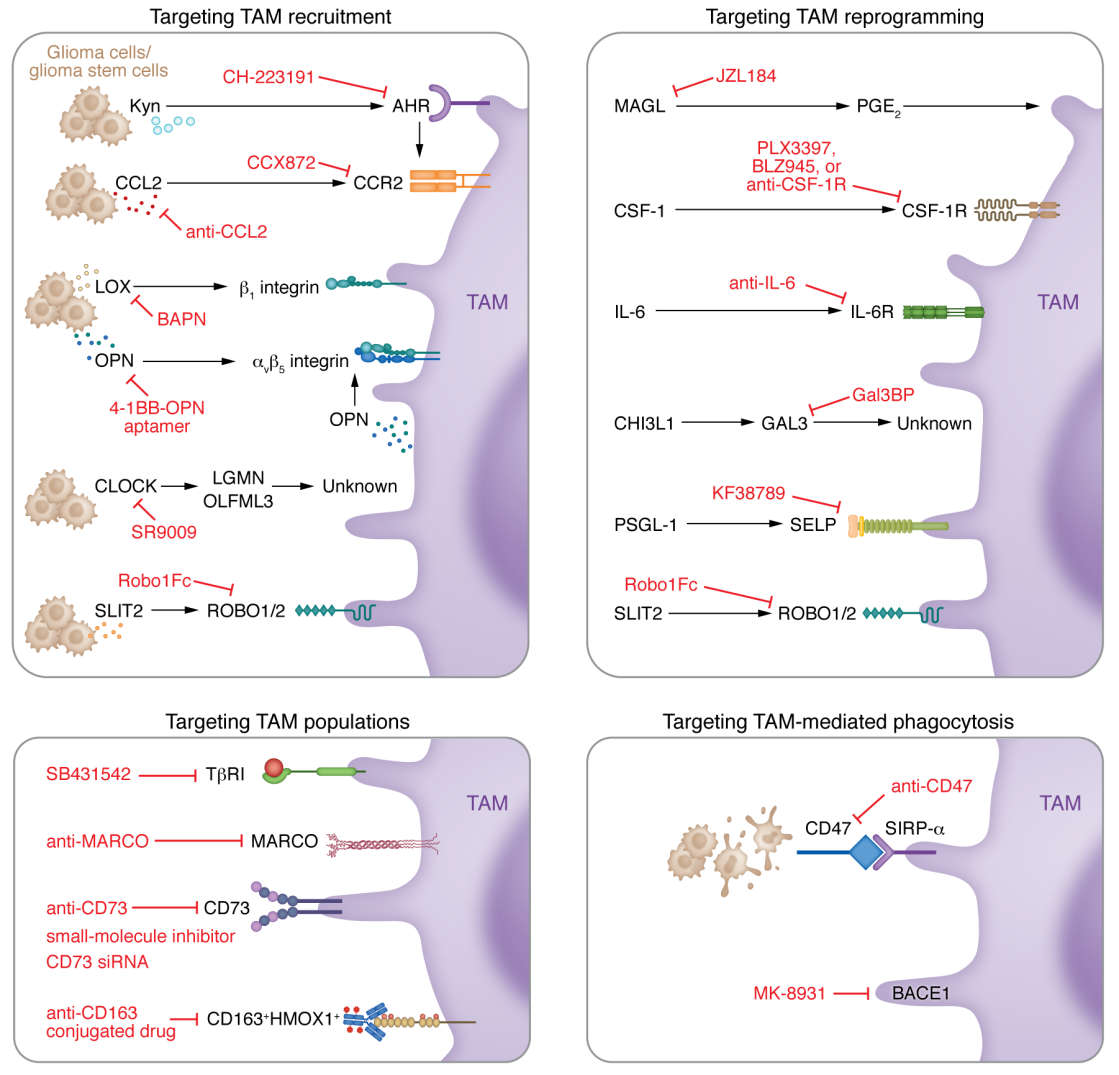
Current TAM-targeted therapeutic approaches in GBM. Depending on the working mechanisms, strategies for targeting TAMs include (a) targeting TAM recruitment; (b) targeting TAM immunosuppressive reprogramming; (c) targeting new TAM subpopulations; and (d) targeting TAM-mediated phagocytosis. The key targets and associated drug candidates are indicated. 4-1BB, TNF receptor superfamily member 9; AHR, aryl hydrocarbon receptor; BACE1, β-site amyloid precursor protein–cleaving enzyme 1; BAPN, β-aminopropionitrile; CCR2, C-C motif chemokine receptor 2; CHI3L1, chitinase-3–like 1; CLOCK, circadian locomotor output cycles protein kaput; CSF-1, colony-stimulating factor 1; CSF-1R, CSF-1 receptor; GAL3, galectin-3; Gal3BP, galectin-3–binding protein; HMOX1, heme oxygenase 1; Kyn, kynurenine; LGMN, legumain; LOX, lysyl oxidase; MAGL, monoacylglycerol lipase; MARCO, macrophage receptor with collagenous structure; OLFML3, olfactomedin-like 3; OPN, osteopontin; PGE_2_, prostaglandin E_2_; PSGL-1, P-selectin glycoprotein ligand-1; ROBO1/2, roundabout receptor 1/2; SELP, P-selectin; SIRPα, signal-regulatory protein-α; SLIT2, slit guidance ligand 2; TβRI, TGF-β receptor type I.

TAMs are a prominent population of immune cells in the GBM TME that play a critical role in supporting tumor progression and inducing immunosuppression (19). Emerging evidence reveals that GBM does not respond to immunotherapy, likely owing, at least in part, to the infiltration of immunosuppressive TAMs (62–64). These findings highlight TAM as a promising therapeutic target for GBM. The following section summarizes current TAM-targeted therapeutic strategies in GBM (Figure 3).

The first approach is to block TAM infiltration by targeting the axes between chemoattractants and their receptors (Figure 3). One of the best-known examples is the CCL2/CCR2 axis. Cancer cell–secreted CCL2 recruits CCR2^+^ myeloid cells (e.g., TAMs and myeloid-derived suppressor cells) into the GBM TME (65, 66). Preclinical data demonstrate that blockade of CCR2 using an antagonist suppresses TAM recruitment and enhances ICI efficacy in GBM mouse models (65, 67). Although further studies are needed to evaluate the antitumor efficiency of CCL2/CCR2 axis blockade in GBM patients, it is worth noting that CCL2 neutralizing antibody (e.g., carlumab) shows a modest effect in patients with prostate cancer (68). If this minimal clinical outcome was observed in GBM, one possible reason is that distinct immune subpopulations may respond differently to CCL2/CCR2 axis inhibition. For example, scRNA-Seq data analysis demonstrates that GBM tumors from *Ccr2*-knockout mice harbor a reduced TAM subpopulation with macrophage signatures (e.g., *TGFBI*, *CLEC12A*, and *FXYD5*), but an increased subpopulation with microglia signatures (e.g., *SALL1*, *TMEM119*, and *P2RY12*) (24), suggesting that the antitumor effect of CCR2 inhibition can be attenuated by increased microglia. Instead of directly inhibiting CCR2, alternative strategies have been developed to suppress the signaling that can potentially induce CCL2/CCR2 axis activation. For example, GBM cell–derived kynurenine would activate the aryl hydrocarbon receptor (AHR) in TAMs, which, in turn, upregulates CCR2, thus promoting TAM infiltration and tumor growth (66). Inhibition of AHR with the antagonist CH-223191 suppresses CCL2-induced TAM infiltration and tumor growth (66). In addition to the CCL2/CCR2 axis, other studies have demonstrated additional targetable chemokine-receptor pairs, such as osteopontin (OPN)/α_v_β_5_ integrin (69), lysyl oxidase (LOX)/ β_1_ integrin (40), and slit guidance ligand 2 (SLIT2)/ROBO1/2 (70), in GBM. Therapeutically, inhibiting these chemokine-receptor pairs using either 4-1BB–OPN bispecific aptamers (69), the LOX inhibitor β-aminopropionitrile or neutralizing antibody (40), or the SLIT2-trapping protein Robo1Fc (70) significantly inhibits macrophage infiltration and tumor growth in GBM mouse models. Moreover, these treatments may improve the antitumor efficiency of ICIs and conventional therapies. For example, the antitumor effect of Robo1Fc was further improved by its combination with anti–PD-1 and anti–4-1BB therapies (70). Mechanistic studies have shown that the chemotactic activity of SLIT2 is regulated by ROBO1/2-mediated PI3Kγ activation in macrophages (70). Consequently, inhibition of PI3Kγ prevents accumulation of TAMs in the GBM TME and elevates the antitumor effect of temozolomide in GBM (71). Moreover, recent studies have shown that overexpression of the circadian regulator CLOCK in GSCs triggers the infiltration of microglia into the GBM TME via transcriptional upregulation of olfactomedin-like 3 (OLFML3) and legumain (LGMN). Inhibition of the axis between CLOCK and its transcriptional targets OLFML3 and LGMN impairs GBM tumor growth and microglial infiltration (25, 44). However, further studies are needed to identify OLFML3 and LGMN receptors on microglia in the GBM TME.

The second strategy is to target TAM immunosuppressive reprogramming (Figure 3). Targeting CSF-1R with its inhibitors (e.g., PLX3397 and BLZ945) can either deplete TAMs or inhibit TAM immunosuppressive polarization in solid tumors, including GBM (24, 72–74). Interestingly, BLZ945 treatment in GBM mouse models fails to deplete TAMs but impairs their functional polarization (75). While CSF-1R inhibition effectively suppresses tumor progression, GBM cells acquire resistance to BLZ945 after long-term treatment (76). Mechanistically, prolonged CSF-1R inhibitor treatment leads to insulin-like growth factor 1 (IGF-1) secretion into the TME via activation of the STAT6/NFAT signaling pathway in TAMs. As a result, the secreted IGF-1 promotes tumor growth by activating the IGF-1R/PI3K pathway in GBM cells. Targeting of the IGF-1R/PI3K signaling in GBM cells using the IGF-1R inhibitor OSI906 and the PI3K inhibitor BKM120, and blocking of the STAT6/NFAT signaling in TAMs using the STAT6 inhibitor AS1517499 and the NFAT-calcineurin inhibitor FK506, resensitize GBM to BLZ945 treatment (76). Moreover, BLZ945 treatment enhances the initial response of GBM to radiotherapy (29) and improves the antitumor efficiency of anti–PD-1 (nivolumab) therapy by blocking CD163^+^ macrophage immunosuppressive polarization (77). Given these encouraging findings in preclinical models and the fact that no clinical benefits were achieved with PLX3397 treatment in GBM patients (21), the results of clinical trials testing novel therapeutic strategies with CSF-1R inhibition combined with IGF-1R/PI3K pathway inhibition, radiotherapy, or immunotherapy are highly anticipated. Alternative therapeutic strategies for manipulating TAM immunosuppressive polarization include anti–IL-6, the SLIT2 ligand trap protein Robo1Fc, the P-selectin inhibitor KF38789, the monoacylglycerol lipase inhibitor JZL184, 4-1BB–OPN aptamer, the β-site amyloid precursor protein–cleaving enzyme 1 (BACE1) inhibitor MK-8931, and galectin-3–binding protein mimetic peptide (12, 69, 70, 78–82). Moreover, a drug consisting of immunostimulatory macrophage extracellular vesicles loaded with the chemical excitation source CPPO (C), the photosensitizer Ce6 (C), and the hydrophilic hypoxia-activated prodrug AQ4N (A) (altogether referred to as CCA) has been developed. CCA exhibits a potent effect to reprogram TAMs and inhibit tumor progression in GBM mouse models (83). Since TAMs are immunosuppressive cells, reprogramming TAMs may enable enhancement of ICI efficiency. Indeed, targeting of TAM reprogramming via IL-6 inhibition with CD40 stimulation (84), SLIT2 inhibition (70), and MAGL inhibition (81) exhibits robust synergy with ICIs (e.g., anti–PD-1, anti-CTLA4, and anti–4-1BB) in preclinical GBM models. Together, these findings highlight that targeting of TAM immunosuppressive reprogramming is a promising strategy that not only inhibits tumor growth but may also improve the antitumor efficiency of ICIs and conventional therapies in GBM.

The third strategy is to target TAM-mediated phagocytosis (Figure 3). Apart from regulating antitumor immunity, TAMs have the ability to directly capture and eliminate cancer cells through phagocytosis (85, 86). However, cancer cells often overexpress CD47, a “don’t eat me” signal that helps cancer cells evade TAM-mediated phagocytosis by interacting with its receptor SIRPα on TAMs (9). Depleting CD47 in GBM cells significantly increases macrophage phagocytosis and inhibits GBM tumor growth (87), indicating the therapeutic potential of targeting the CD47/SIRPα axis in GBM patients. The anti-CD47 strategy is under investigation in clinical trials for solid tumors and hematological malignancies (e.g., ClinicalTrials.gov NCT02953782 and NCT02890368) (88, 89). Notably, monotherapeutic anti-CD47 antibodies show a minor effect on glioma growth in murine models (90) but induce hematological toxicity (91). In contrast, preclinical studies with humanized anti-CD47 antibodies have shown promising antitumor effects in pediatric glioma patient-derived xenograft models (85). These findings highlight that additional approaches will be needed to improve the efficacy and safety of anti-CD47 therapy in GBM. Emerging evidence demonstrates that the antitumor effect of anti-CD47 therapy can be enhanced when it is combined with temozolomide and anti–PD-1 treatments (90), carnitine palmitoyltransferase 1 inhibitor (etomoxir) (92), or autophagy depletion (93) in GBM mouse models. In line with enhancing the antitumor effect of anti-CD47 therapy, an oncolytic herpes virus has been generated to avoid infusion toxicities and increase the blood-brain barrier–penetrating efficiency of anti-CD47, which exhibits superior tumor cytotoxicity in GBM (94). In line with anti-CD47, targeting SIRPα is a potential therapeutic strategy since recent evidence demonstrates that anti-SIRPα nanobodies can penetrate into GBM tumors in mice (95). Besides targeting the CD47/SIRPα axis, pharmacological inhibition of BACE1 with the inhibitor MK-8931 promotes TAM-mediated phagocytosis of GSCs and impairs GBM progression in vivo (79). Together, these findings demonstrate that targeting of TAM-mediated phagocytosis exhibits promising therapeutic potential for GBM.

The final strategy is to target the newly identified TAM subpopulations (Figure 3). For example, targeting CD73^hi^ macrophages via depletion of CD73 in CD73^−/−^ mice extends the survival of GBM-bearing mice, and this effect is further improved when combined with anti–PD-1 and anti-CTLA4 therapies (62). Cancer treatment with anti-CD73 antibodies and CD73 small-molecule inhibitors has gained promising results in preclinical and early clinical trials (96, 97). However, such antitumor effect has not been compelling in a preclinical murine model of glioma (98). The newly identified MARCO^hi^ macrophage subpopulation can promote GBM tumor growth in vitro and in vivo. Treatment with anti-MARCO antibodies inhibits mesenchymal differentiation and stemness of GSCs (61). HGG-AM is a newly identified microglia population that can be activated by glioma cell–derived TGF-β1 via TGF-β receptor type I (TβRI) on microglia. Reciprocally, these activated HGG-AM produce IL-1β to promote GSC proliferation and tumor growth. Inhibition of TβRI using its inhibitor SB431542 diminishes HGG-AM density and impairs tumor growth in a GBM mouse model (58). CD163^+^HMOX1^+^ microglia are another newly identified microglia subpopulation in GBM, and depletion of this microglia population reduces IL-10 production, which, in turn, upregulates granzyme B in T cells via the JAK/STAT pathway. Treatment with the JAK1/2 inhibitor ruxolitinib in a GBM patient boosted T cell activation by reducing immunosuppressive myeloid cells, and the patient is still alive about 2 years after ruxolitinib treatment (59). Together, these findings suggest that these newly identified TAM subpopulations (CD73^hi^ macrophages, MARCO^hi^ TAMs, HGG-AM, and CD163^+^HMOX1^+^ microglia) are promising therapeutic targets for GBM patients.

## Conclusion

TAMs are highly infiltrated in GBM tumors and substantially contribute to tumor progression, immunosuppression, and treatment resistance (12, 24, 40). Understanding the heterogeneity and functional plasticity of TAMs is crucial for developing context-dependent therapeutic strategies for GBM patients. Although classical methods, such as fluorescence-activated cell sorting and immunofluorescence, can distinguish functional TAMs based on well-known phenotypic markers, they are not sufficient to characterize TAM heterogeneity in the GBM TME (12, 33). In contrast, single-cell technologies have several advantages. For example, they can offer an excellent opportunity to identify novel TAM subpopulations and functional states in GBM (24, 99). These new TAM subpopulations are crucial for GBM progression, although they may account for only a small proportion of myeloid cells in the TME (24, 33, 99, 100). Studies integrating scRNA-Seq and functional validations demonstrate that these TAM subpopulations are functional and druggable targets (38, 62, 101). Specifically, these TAM subpopulations may preferentially secrete specific cytokines (e.g., IL-10 from CD163^+^HMOX1^+^ microglia) to induce immunosuppression, and targeting these cytokines and their relevant molecular pathways holds great therapeutic potential in GBM (20, 59). Moreover, they can provide transcriptional information for tracing the ontogeny and distribution of TAMs in GBM (102), which, in turn, produces additional markers and location information to distinguish macrophages from microglia (19, 33). Finally, they can enable researchers to generate transcriptional networks among different cell populations and subpopulations and to have an integrated view of how TAMs shape an immunosuppressive TME in GBM (24, 101). For instance, scRNA-Seq data analysis reveals that macrophages and microglia compete with each other, and macrophage depletion leads to increased microglia infiltration in the hypoxic TME (24).

Despite the advantages of single-cell technologies for characterizing TAM heterogeneity and plasticity, many challenges remain regarding how to maximize these technologies to understand the nature of TAMs in GBM and translate these findings into the clinic (103, 104). First, single-cell technologies are not efficient in systemically dissecting TAM function in vivo. Rigorous functional validations are needed to interrogate how specific subpopulations and functional states of TAMs connect to GBM cells and other immune cells in the TME and contribute to GBM progression and immunosuppression (25, 38, 40, 44, 61, 62). Second, scRNA-Seq may not be able to define some key molecular states (e.g., epigenomic and metabolic states) of TAMs under specific GBM TME (19, 99). An alternative strategy is to integrate scRNA-Seq and epigenomic/metabolomic profiling, which will provide additional information to characterize TAM heterogeneity (43, 105). The third challenge is a technical issue regarding clustering algorithms (106). As a rapidly growing field, many computational methods have been developed to define TAM populations and functional states based on scRNA-Seq data (104). However, it is still unclear which is the best approach to characterize the dynamic and heterogeneous TAMs in GBM (106). Moreover, the strict sample preparation requirement for large-scale scRNA-Seq analysis on human GBM tumor tissues is still challenging (107, 108). Finally, the spatial distribution of TAMs can generate additional heterogeneity, since specific local niches are able to affect TAM distribution and functional polarization (109, 110). For example, IBA1^+^TMEM119^–^CXCL3^+^ macrophages are predominantly located at perinecrotic areas and express genes involved in inflammatory program and glycolysis, whereas IBA1^+^TMEM119^+^CCL4^+^ microglia are enriched at the tumor-brain interface and express chemoattractant-encoding genes involved in T cell recruitment (110). Characterization of TAM spatial distribution and identification of the context-dependent niche signals that drive TAM activation and polarization are two remaining challenges. Integrating single-cell and spatial transcriptomics will provide an opportunity to characterize this spatial TAM heterogeneity (38, 111–113). It is expected that continuing research to address these challenges, followed by rigorous functional studies, will uncover the nature of TAMs and identify novel TAM-targeted immunotherapy for GBM patients in the near future.

## Version 1 01/03/2023

Electronic publication

## References

[1] Roda E, Bottone MG (2022). Editorial: Brain cancers: new perspectives and therapies. Front Neurosci.

[2] Qazi MA (2017). Intratumoral heterogeneity: pathways to treatment resistance and relapse in human glioblastoma. Ann Oncol.

[3] Dunn GP (2012). Emerging insights into the molecular and cellular basis of glioblastoma. Genes Dev.

[4] Puram SV (2018). Single cell RNA-seq highlights a role for a partial EMT in head and neck cancer. Mol Cell Oncol.

[5] Patel AP (2014). Single-cell RNA-seq highlights intratumoral heterogeneity in primary glioblastoma. Science.

[6] Doucette T (2013). Immune heterogeneity of glioblastoma subtypes: extrapolation from the cancer genome atlas. Cancer Immunol Res.

[7] Wang Q (2017). Tumor evolution of glioma-intrinsic gene expression subtypes associates with immunological changes in the microenvironment. Cancer Cell.

[8] Quail DF, Joyce JA (2013). Microenvironmental regulation of tumor progression and metastasis. Nat Med.

[9] Chen P (2021). Cancer stemness meets immunity: from mechanism to therapy. Cell Rep.

[10] Chen P, Dey P (2022). Co-dependencies in the tumor immune microenvironment. Oncogene.

[11] Da Ros M (2018). Glioblastoma chemoresistance: the double play by microenvironment and blood-brain barrier. Int J Mol Sci.

[12] Pang L (2022). Pharmacological targeting of the tumor-immune symbiosis in glioblastoma. Trends Pharmacol Sci.

[13] Xuan W (2021). Circadian regulation of cancer cell and tumor microenvironment crosstalk. Trends Cell Biol.

[14] Pang L (2022). Mechanism and therapeutic potential of tumor-immune symbiosis in glioblastoma. Trends Cancer.

[15] Sanmamed MF, Chen L (2018). A paradigm shift in cancer immunotherapy: from enhancement to normalization. Cell.

[16] Sharma P, Allison JP (2015). Immune checkpoint targeting in cancer therapy: toward combination strategies with curative potential. Cell.

[17] de Groot J (2020). Window-of-opportunity clinical trial of pembrolizumab in patients with recurrent glioblastoma reveals predominance of immune-suppressive macrophages. Neuro Oncol.

[18] Klemm F (2020). Interrogation of the microenvironmental landscape in brain tumors reveals disease-specific alterations of immune cells. Cell.

[19] Xuan W (2021). Context-dependent glioblastoma-macrophage/microglia symbiosis and associated mechanisms. Trends Immunol.

[20] Simonds EF (2021). Deep immune profiling reveals targetable mechanisms of immune evasion in immune checkpoint inhibitor-refractory glioblastoma. J Immunother Cancer.

[21] Butowski N (2016). Orally administered colony stimulating factor 1 receptor inhibitor PLX3397 in recurrent glioblastoma: an Ivy Foundation Early Phase Clinical Trials Consortium phase II study. Neuro Oncol.

[22] Neftel C (2019). An integrative model of cellular states, plasticity, and genetics for glioblastoma. Cell.

[23] Friebel E (2020). Single-cell mapping of human brain cancer reveals tumor-specific instruction of tissue-invading leukocytes. Cell.

[24] Pombo Antunes AR (2021). Single-cell profiling of myeloid cells in glioblastoma across species and disease stage reveals macrophage competition and specialization. Nat Neurosci.

[25] Xuan W (2022). Circadian regulator CLOCK drives immunosuppression in glioblastoma. Cancer Immunol Res.

[26] Muller A (2015). Resident microglia, and not peripheral macrophages, are the main source of brain tumor mononuclear cells. Int J Cancer.

[27] Youshani AS (2019). Non-myeloablative busulfan chimeric mouse models are less pro-inflammatory than head-shielded irradiation for studying immune cell interactions in brain tumours. J Neuroinflammation.

[28] Brandenburg S (2020). Distinction of microglia and macrophages in glioblastoma: close relatives, different tasks?. Int J Mol Sci.

[29] Akkari L (2020). Dynamic changes in glioma macrophage populations after radiotherapy reveal CSF-1R inhibition as a strategy to overcome resistance. Sci Transl Med.

[30] Friedrich M (2021). Tryptophan metabolism drives dynamic immunosuppressive myeloid states in IDH-mutant gliomas. Nat Cancer.

[31] Haage V (2019). Comprehensive gene expression meta-analysis identifies signature genes that distinguish microglia from peripheral monocytes/macrophages in health and glioma. Acta Neuropathol Com.

[32] Li Q (2018). Expression of *Tmem119*/*Sall1* and *Ccr2*/*CD69* in FACS-sorted microglia- and monocyte/macrophage-enriched cell populations after intracerebral hemorrhage. Front Cell Neurosci.

[33] Ochocka N (2021). Single-cell RNA sequencing reveals functional heterogeneity of glioma-associated brain macrophages. Nat Commun.

[34] Woolf Z (2021). Single-cell image analysis reveals a protective role for microglia in glioblastoma. Neurooncol Adv.

[35] Chen Z (2017). Cellular and molecular identity of tumor-associated macrophages in glioblastoma. Cancer Res.

[36] Bowman RL (2016). Macrophage ontogeny underlies differences in tumor-specific education in brain malignancies. Cell Rep.

[37] Chen Z (2019). Intravital 2-photon imaging reveals distinct morphology and infiltrative properties of glioblastoma-associated macrophages. Proc Natl Acad Sci U S A.

[38] Abdelfattah N (2022). Single-cell analysis of human glioma and immune cells identifies S100A4 as an immunotherapy target. Nat Commun.

[39] Brennan CW (2013). The somatic genomic landscape of glioblastoma. Cell.

[40] Chen P (2019). Symbiotic macrophage-glioma cell interactions reveal synthetic lethality in PTEN-null glioma. Cancer Cell.

[41] Melhem JM Updates in IDH-wildtype glioblastoma. Neurotherapeutics.

[42] Schmitt MJ (2021). Phenotypic mapping of pathologic cross-talk between glioblastoma and innate immune cells by synthetic genetic tracing. Cancer Discov.

[43] Gangoso E (2021). Glioblastomas acquire myeloid-affiliated transcriptional programs via epigenetic immunoediting to elicit immune evasion. Cell.

[44] Chen P (2020). Circadian regulator CLOCK recruits immune-suppressive microglia into the GBM tumor microenvironment. Cancer Discov.

[45] Hu X (2020). Microglia/macrophage polarization: fantasy or evidence of functional diversity?. J Cereb Blood Flow Metab.

[46] Martinez FO, Gordon S (2014). The M1 and M2 paradigm of macrophage activation: time for reassessment. F1000Prime Rep.

[47] Cheng N (2021). Targeting tumor-associated macrophages as an antitumor strategy. Biochem Pharmacol.

[48] Mantovani A (2004). The chemokine system in diverse forms of macrophage activation and polarization. Trends Immunol.

[49] Chen P (2015). Role of macrophages in Wallerian degeneration and axonal regeneration after peripheral nerve injury. Acta Neuropathol.

[50] Shapouri-Moghaddam A (2018). Macrophage plasticity, polarization, and function in health and disease. J Cell Physiol.

[51] Mills CD (2000). M-1/M-2 macrophages and the Th1/Th2 paradigm. J Immunol.

[52] Muller S (2017). Single-cell profiling of human gliomas reveals macrophage ontogeny as a basis for regional differences in macrophage activation in the tumor microenvironment. Genome Biol.

[53] Xue J (2014). Transcriptome-based network analysis reveals a spectrum model of human macrophage activation. Immunity.

[54] Martinez-Lage M (2019). Immune landscapes associated with different glioblastoma molecular subtypes. Acta Neuropathol Commun.

[55] Miyazaki T (2020). Infiltration of CD163-positive macrophages in glioma tissues after treatment with anti-PD-L1 antibody and role of PI3Kγ inhibitor as a combination therapy with anti-PD-L1 antibody in in vivo model using temozolomide-resistant murine glioma-initiating cells. Brain Tumor Pathol.

[56] Murray PJ (2014). Macrophage activation and polarization: nomenclature and experimental guidelines. Immunity.

[57] Yeo AT (2022). Single-cell RNA sequencing reveals evolution of immune landscape during glioblastoma progression. Nat Immunol.

[58] Liu H (2021). Pro-inflammatory and proliferative microglia drive progression of glioblastoma. Cell Rep.

[59] Ravi VM (2022). T-cell dysfunction in the glioblastoma microenvironment is mediated by myeloid cells releasing interleukin-10. Nat Commun.

[60] Khalsa JK (2020). Immune phenotyping of diverse syngeneic murine brain tumors identifies immunologically distinct types. Nat Commun.

[61] Sa JK (2020). Transcriptional regulatory networks of tumor-associated macrophages that drive malignancy in mesenchymal glioblastoma. Genome Biol.

[62] Goswami S (2020). Immune profiling of human tumors identifies CD73 as a combinatorial target in glioblastoma. Nat Med.

[63] Aslan K (2020). Heterogeneity of response to immune checkpoint blockade in hypermutated experimental gliomas. Nat Commun.

[64] Zhao J (2019). Immune and genomic correlates of response to anti-PD-1 immunotherapy in glioblastoma. Nat Med.

[65] Flores-Toro JA (2020). CCR2 inhibition reduces tumor myeloid cells and unmasks a checkpoint inhibitor effect to slow progression of resistant murine gliomas. Proc Natl Acad Sci U S A.

[66] Takenaka MC (2019). Control of tumor-associated macrophages and T cells in glioblastoma via AHR and CD39. Nat Neurosci.

[67] Shao ZH (2022). Molecular insights into ligand recognition and activation of chemokine receptors CCR2 and CCR3. Cell Discov.

[68] Pienta KJ (2013). Phase 2 study of carlumab (CNTO 888), a human monoclonal antibody against CC-chemokine ligand 2 (CCL2), in metastatic castration-resistant prostate cancer. Invest New Drugs.

[69] Wei J (2019). Osteopontin mediates glioblastoma-associated macrophage infiltration and is a potential therapeutic target. J Clin Invest.

[70] Geraldo LH (2021). SLIT2/ROBO signaling in tumor-associated microglia and macrophages drives glioblastoma immunosuppression and vascular dysmorphia. J Clin Invest.

[71] Li J (2021). PI3Kγ inhibition suppresses microglia/TAM accumulation in glioblastoma microenvironment to promote exceptional temozolomide response. Proc Natl Acad Sci U S A.

[72] Mantovani A (2017). Tumour-associated macrophages as treatment targets in oncology. Nat Rev Clin Oncol.

[73] Noy R, Pollard JW (2014). Tumor-associated macrophages: from mechanisms to therapy. Immunity.

[74] Pittet MJ (2022). Clinical relevance of tumour-associated macrophages. Nat Rev Clin Oncol.

[75] Pyonteck SM (2013). CSF-1R inhibition alters macrophage polarization and blocks glioma progression. Nat Med.

[76] Quail DF (2016). The tumor microenvironment underlies acquired resistance to CSF-1R inhibition in gliomas. Science.

[77] Cui X (2020). Dissecting the immunosuppressive tumor microenvironments in glioblastoma-on-a-chip for optimized PD-1 immunotherapy. Elife.

[78] Wang QR (2018). Vascular niche IL-6 induces alternative macrophage activation in glioblastoma through HIF-2 alpha. Nat Commun.

[79] Zhai K (2021). Pharmacological inhibition of BACE1 suppresses glioblastoma growth by stimulating macrophage phagocytosis of tumor cells. Nat Cancer.

[80] Yeini E (2021). P-selectin axis plays a key role in microglia immunophenotype and glioblastoma progression. Nat Commun.

[81] Yin J (2020). ARS2/MAGL signaling in glioblastoma stem cells promotes self-renewal and M2-like polarization of tumor-associated macrophages. Nat Commun.

[82] Chen A (2021). Chitinase-3-like 1 protein complexes modulate macrophage-mediated immune suppression in glioblastoma. J Clin Invest.

[83] Wang X (2022). Exploration and functionalization of M1-macrophage extracellular vesicles for effective accumulation in glioblastoma and strong synergistic therapeutic effects. Signal Transduct Target Ther.

[84] Yang F (2021). Synergistic immunotherapy of glioblastoma by dual targeting of IL-6 and CD40. Nat Commun.

[85] Gholamin S (2017). Disrupting the CD47-SIRP alpha anti-phagocytic axis by a humanized anti-CD47 antibody is an efficacious treatment for malignant pediatric brain tumors. Sci Transl Med.

[86] Hutter G (2019). Microglia are effector cells of CD47-SIRPα antiphagocytic axis disruption against glioblastoma. Proc Natl Acad Sci U S A.

[87] Ma D (2019). Extracellular matrix protein tenascin c increases phagocytosis mediated by CD47 loss of function in glioblastoma. Cancer Res.

[88] Querfeld C (2021). Intralesional TTI-621, a novel biologic targeting the innate immune checkpoint CD47, in patients with relapsed or refractory mycosis fungoides or Sezary syndrome: a multicentre, phase 1 study. Lancet Haematol.

[89] Kaur S (2020). Preclinical and clinical development of therapeutic antibodies targeting functions of CD47 in the tumor microenvironment. Antib Ther.

[90] von Roemeling CA (2020). Therapeutic modulation of phagocytosis in glioblastoma can activate both innate and adaptive antitumour immunity. Nat Commun.

[91] Sikic BI (2019). First-in-human, first-in-class phase I trial of the anti-CD47 antibody Hu5F9-G4 in patients with advanced cancers. J Clin Oncol.

[92] Jiang N (2022). Fatty acid oxidation fuels glioblastoma radioresistance with CD47-mediated immune evasion. Nat Commun.

[93] Zhang X (2018). Disrupting CD47-SIRPalpha axis alone or combined with autophagy depletion for the therapy of glioblastoma. Carcinogenesis.

[94] Xu B (2021). An oncolytic virus expressing a full-length antibody enhances antitumor innate immune response to glioblastoma. Nat Commun.

[95] De Vlaminck K (2021). Imaging of glioblastoma tumor-associated myeloid cells using nanobodies targeting signal regulatory protein alpha. Front Immunol.

[96] Antonioli L (2017). Switching off CD73: a way to boost the activity of conventional and targeted antineoplastic therapies. Drug Discov Today.

[97] Perrot I (2019). Blocking antibodies targeting the CD39/CD73 immunosuppressive pathway unleash immune responses in combination cancer therapies. Cell Rep.

[98] Ott M (2020). Profiling of patients with glioma reveals the dominant immunosuppressive axis is refractory to immune function restoration. JCI Insight.

[99] Bian Z (2020). Deciphering human macrophage development at single-cell resolution. Nature.

[100] Benmamar-Badel A (2020). Protective microglial subset in development, aging, and disease: lessons from transcriptomic studies. Front Immunol.

[101] Hara T (2021). Interactions between cancer cells and immune cells drive transitions to mesenchymal-like states in glioblastoma. Cancer Cell.

[102] Van Hove H (2019). A single-cell atlas of mouse brain macrophages reveals unique transcriptional identities shaped by ontogeny and tissue environment. Nat Neurosci.

[103] Lahnemann D (2020). Eleven grand challenges in single-cell data science. Genome Biol.

[104] Kharchenko PV (2021). The triumphs and limitations of computational methods for scRNA-seq. Nat Methods.

[105] Geeraerts X (2021). Macrophages are metabolically heterogeneous within the tumor microenvironment. Cell Rep.

[106] Kiselev VY (2019). Challenges in unsupervised clustering of single-cell RNA-seq data. Nat Rev Genet.

[107] Chu T (2022). Cell type and gene expression deconvolution with BayesPrism enables Bayesian integrative analysis across bulk and single-cell RNA sequencing in oncology. Nat Cancer.

[108] Denisenko E (2020). Systematic assessment of tissue dissociation and storage biases in single-cell and single-nucleus RNA-seq workflows. Genome Biol.

[109] Sankowski R (2019). Mapping microglia states in the human brain through the integration of high-dimensional techniques. Nat Neurosci.

[110] Yin W (2022). A map of the spatial distribution and tumour-associated macrophage states in glioblastoma and grade 4 IDH-mutant astrocytoma. J Pathol.

[111] Ahmed R (2022). Single-cell RNA sequencing with spatial transcriptomics of cancer tissues. Int J Mol Sci.

[112] Longo SK (2021). Integrating single-cell and spatial transcriptomics to elucidate intercellular tissue dynamics. Nat Rev Genet.

[113] Ravi VM (2022). Spatially resolved multi-omics deciphers bidirectional tumor-host interdependence in glioblastoma. Cancer Cell.

